# Supplementary material for: A convolutional neural network STIFMap reveals associations between stromal stiffness and EMT in breast cancer
Source: Nat Commun. 2023 Jun 15;14:3561. doi: 10.1038/s41467-023-39085-1 (PMC10272194; doi:10.1038/s41467-023-39085-1)
Supplement: Supplementary file 1 — Supplementary Information [file 41467_2023_39085_MOESM1_ESM.pdf]

A convolutional neural network STIFMap reveals associations between stromal stiffness and EMT in breast cancer

Connor Stashko[1,2], Mary-Kate Hayward[1,2,*], Jason J. Northey[1,2,*], Neil Pearson, Alastair J. Ironside[3], Johnathon N. Lakins[1,2], Roger Oria [1,2], Marie-Anne Goyette[4], Lakyn Mayo[5], Hege G. Russnes[6,7], E. Shelley Hwang[8], Matthew L. Kutys[5,9], Kornelia Polyak[4], Valerie M. Weaver[1,2,9,10,]**

[1]Department of Surgery, University of California, San Francisco, California, USA.
[2]Center for Bioengineering and Tissue Regeneration, University of California, San Francisco, San Francisco, CA, USA.
[3]Department of Pathology, Western General Hospital, NHS Lothian, Edinburgh, UK.
[4]Department of Medical Oncology, Dana-Farber Cancer Institute, Boston, MA, USA.
[5]Department of Cell and Tissue Biology, School of Dentistry, University of California, San Francisco, San Francisco, CA, USA.
[6]Department of Pathology and Department of Cancer Genetics, Institute for Cancer Research, Oslo University Hospital, Oslo, Norway.
[7]Institute for Clinical Medicine, University of Oslo, Oslo, Norway.
[8]Department of Surgery, Duke University Medical Center, Durham, NC, USA.
[9]UCSF Helen Diller Comprehensive Cancer Center, University of California, San Francisco, San Francisco, CA, USA.
[10]Department of Radiation Oncology, Eli and Edythe Broad Center of Regeneration Medicine and Stem Cell Research, University of California, San Francisco, San Francisco, CA, USA.
* These authors contributed equally to this work
** **Corresponding Author:**
Valerie M. Weaver
Email: valerie.weaver@ucsf.edu

SUPPLEMENTARY INFORMATION

ITEM NO.	SW-File Name (File Name)	DESCRIPTION	PROCESS	SUPPLIER	PART NUMBER	QTY.	Package Qty	Unit Price ($)	Order Qty	Extended Price ($)
1	Microscope Stage_base	AFM Stage Base	INCLUDED			1				
2	Microscope Stage_X	AFM Stage X Translation	INCLUDED			1				
3	Microscope Stage_Y	AFM Stage Y Transation	INCLUDED			1				
4	Microscope Stage_X_knob	AFM X Knob	INCLUDED			1				
5	Microscope Stage_Y_knob	AFM Y Knob	INCLUDED			1				
6	clamp_screw_91290A19 0	Screws frame_main into frame_clamp	PURCHASED	MCMASTER	91290A190	4	10	7.11	1	7.11
7	clamp_nut_90576A103	Screws frame_main into frame_clamp	PURCHASED	MCMASTER	90576A103	4	100	4.27	1	4.27
8	clamp_washer_93475A2 30	Screws frame_main into frame_clamp	PURCHASED	MCMASTER	93475A230	4	100	1.86	1	1.86
9	X_frame_main	Stage Frame_x	3D PRINTED			1				
10	NEMA 17	Motor	INCLUDED		NEMA 17	2				
11	motor_faceplate	Motor Bracket	3D PRINTED			2				
12	slider	Motor Frame	3D PRINTED			2				
13	wedge_1	Bracket Adjuster_front	3D PRINTED			2				
14	wedge_2	Bracket Adjuster_back	3D PRINTED			2				
15	wedge_spring_5108N27 1	Springs holding Motor Bracket with Motor Frame	PURCHASED	MCMASTER	9044K113	4	3	5.26	2	10.52
16	wedge_pin_m3x36_9159 5A140	Tensioning Pin	PURCHASED	MCMASTER	91595A140	4	25	9.91	1	9.91
17	wedge_screw_92000A0 77	Bracket Adjusting Screws	PURCHASED	MCMASTER	92000A077	4	50	9.94	1	9.94
18	wedge_nut_90591A250	Used with Bracket Adjusting Screws	PURCHASED	MCMASTER	90591A250	4	100	2.33	1	2.33
19	wedge_washer_97310A 111_	Used with Bracket Adjusting Screws	PURCHASED	MCMASTER	97310A111	4	100	2.86	1	2.86
20	slider_pin_91585A389	Alignment Rod	PURCHASED	MCMASTER	91585A389	4	1	4.14	4	16.56
21	motor_screw_91290A111	Screws Motor into Motor Bracket	PURCHASED	MCMASTER	91290A111	4	100	8.71	1	8.71
22	X_frame_clamp	Clamps Stage Frame_x onto AFM Stage	3D PRINTED			1				
23	knob_cap	Knob Adapter	3D PRINTED			2				
24	knob_screw_90044A247	Adapter Screw	PURCHASED	MCMASTER	90044A247	2	5	7.44	1	7.44
25	5mm_hub_9889T106	Motor Coupling	PURCHASED	MCMASTER	9889T106	2	1	16.08	2	32.16
26	8mm_Hub_9889T109	Motor Coupling	PURCHASED	MCMASTER	9889T109	2	1	16.08	2	32.16
27	Acetal_disk_59985K620	Motor Coupling	PURCHASED	MCMASTER	59985K62	2	1	3.22	2	6.44
28	rubber pad_X	Between X_frame_main and X_frame_clamp	INCLUDED			1				
29	Yframe_main	Stage Frame_y	3D PRINTED			1				
30	Yframe_clamp	Clamps Stage Frame_y onto AFM Stage	3D PRINTED			1				
31	rubber pad_Y	Between Y_frame_main and Y_frame_clamp	INCLUDED			1				
32	rollers	Rollers screw into Y_frame_main	PURCHASED	MCMASTER	3659K11	2	1	36.88	2	73.76
									TOTAL	226.03

Supplementary Table 1. AutoAFM Bill of Materials. List of components required to implement an AutoAFM system on an existing MFP 3D Bio AFM (Asylum Research).

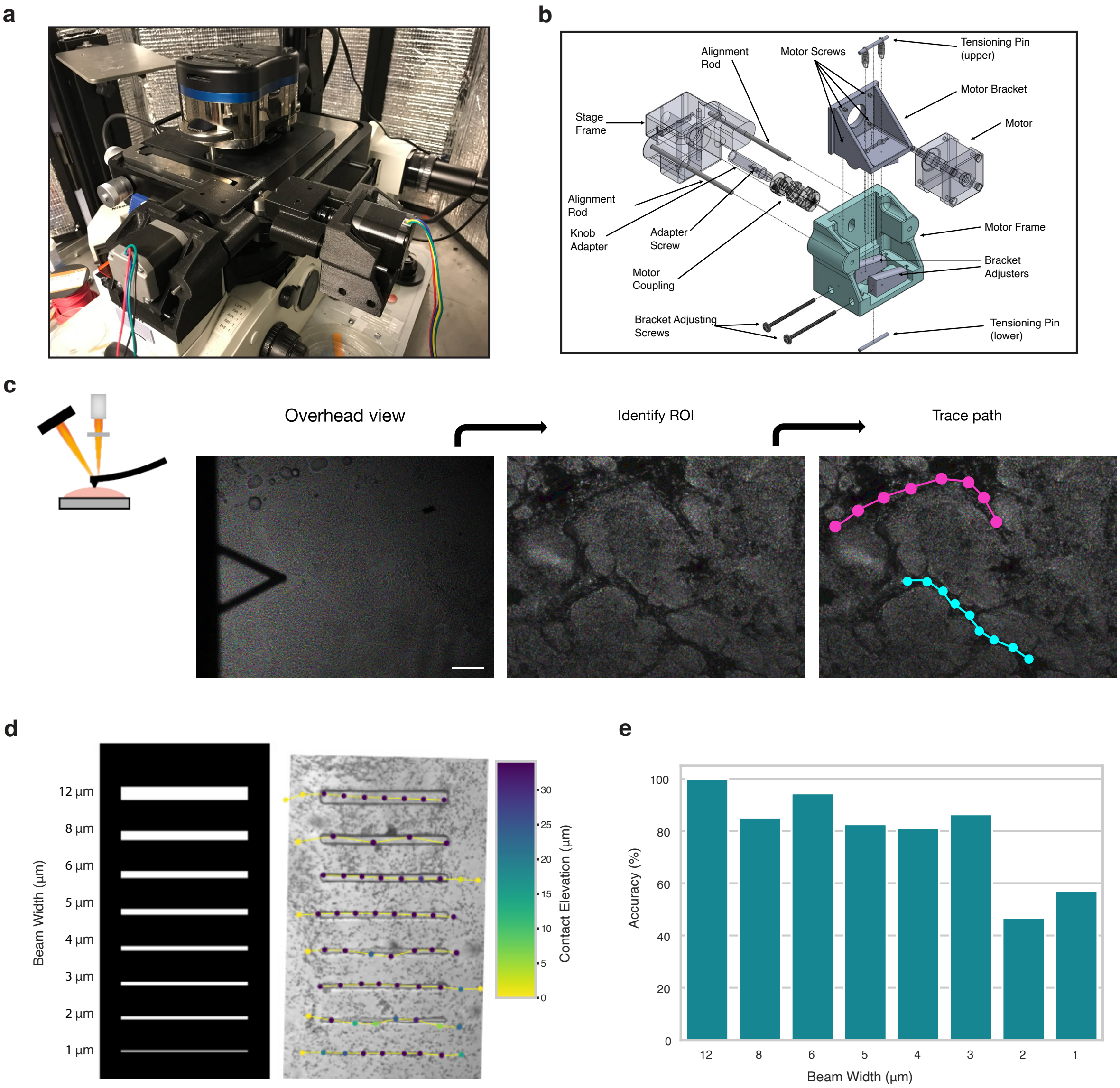

Supplementary Figure 1. AutoAFM Principle and Validation. a, Photo of AutoAFM assembly. **b,** Technical drawing of all main components of the AutoAFM system. **c,** AutoAFM workflow. The AutoAFM workflow was used for generating 200 trace paths. **d,** Overview of PDMS balance beam design (left) and actual fabrication (right) with points overlaid. **e,** Accuracy of AutoAFM movements along each PDMS beam. Scale bar, 100 µm. Source data are provided as a Source Data file.

a

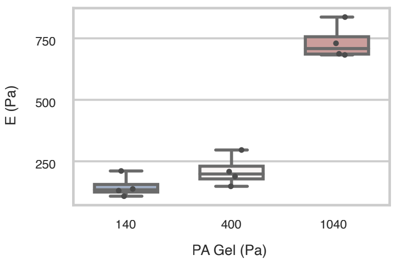

PA Gel Nominal Stiffness (PA)	Actual Stiffness (PA)
140	146.71
400	210.19
1040	733.97

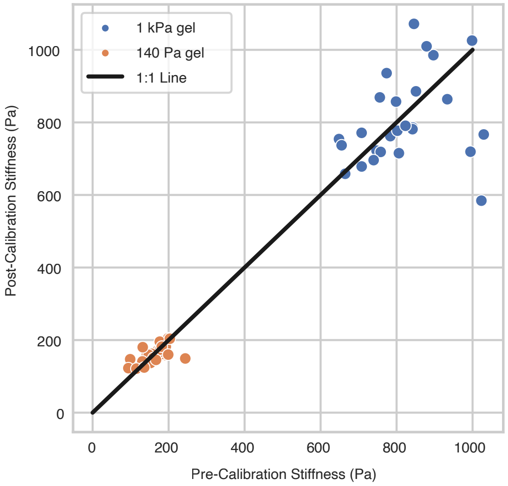

b

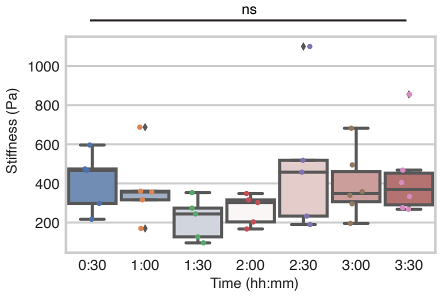

c

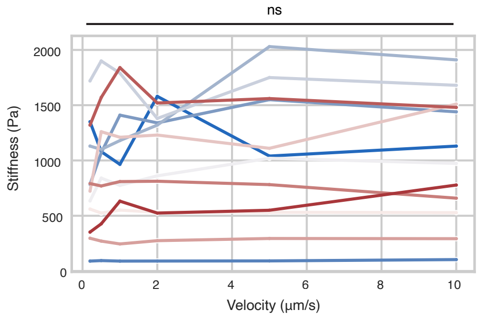

d

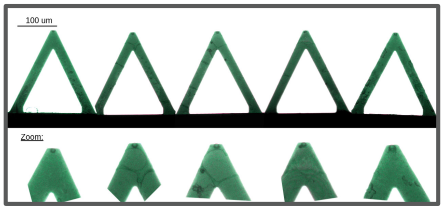

e

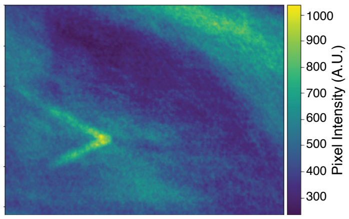

f

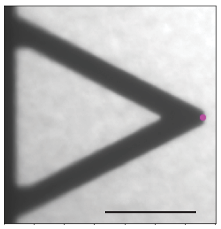

g

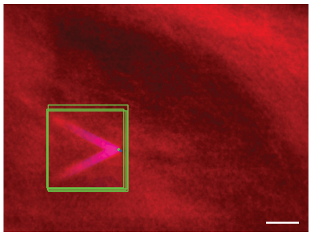

Supplementary Figure 2. AFM Control Experiments. a, Young's Moduli of polyacrylamide (PA) gels of different nominal elasticities measured using shear rheology (left). n = 4 gels of each elasticity. Pre and post-sampling calibration of cantilever tip in PA gels (right). **b,** Time course of the same tissue region probed with AFM every thirty minutes for 3.5 hours (n = 5 positions probed per timepoint) p-value=0.93. **c,** Elasticity of different tissue positions probed with AFM at different velocities. n = 12 positions. **d,** Representative images of AFM cantilevers used. **e,** AFM cantilever artifact in the average image for an AutoAFM scan. **f,** 5 μm ball position on the end of an AFM cantilever. **g,** AFM cantilever ball positions fit onto the AFM artifact from an average image (**e**)**.** Boxes denote 25th to 75th percentile with median line. Whiskers mark the minima and the maxima excluding outliers. Statistical analyses used were performed using two-sided Mann-Whitney U test, ns = non-significant. Scale bar, 100 μm. Source data are provided as a Source Data file.

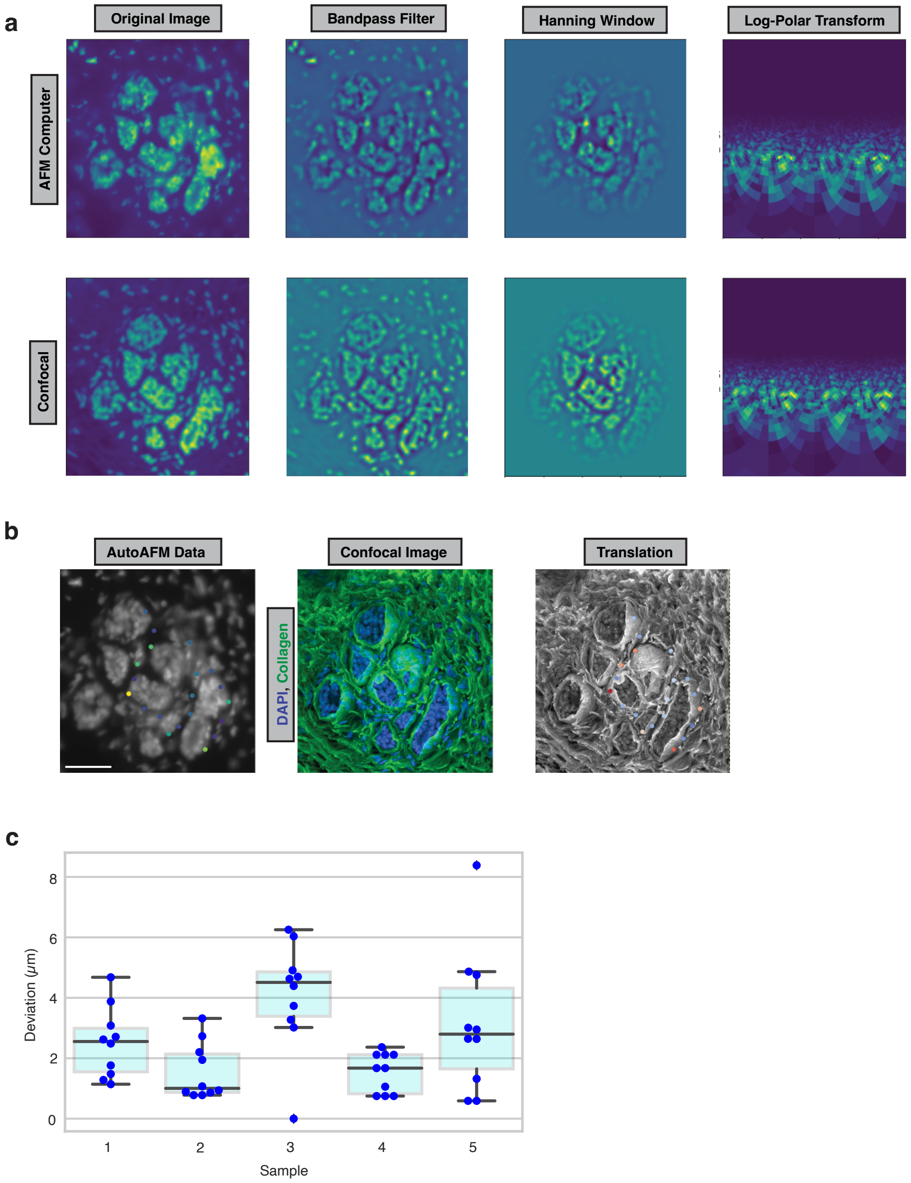

Supplementary Figure 3. Image Stitching and Overlaying. a, Pipeline for Fourier-Mellin Transformation. The confocal DAPI image was downsampled (bottom left) to better resemble the AFM image (top left). Then, both images were processed with a Bandpass Filter, Hanning Window, and Log-Polar Transformation. Translational differences between the final images were converted into scaling and rotation differences in the original images. **b,** Overall translation of AutoAFM data from a low-resolution AFM microscope image onto the high-resolution confocal image. **c,** Deviation in cell positions after transformation. n = 50 cell positions from five samples. Boxes denote 25th to 75th percentile with median line. Whiskers mark the minima and the maxima excluding outliers. Scale bar, 50 μm. Source data are provided as a Source Data file.

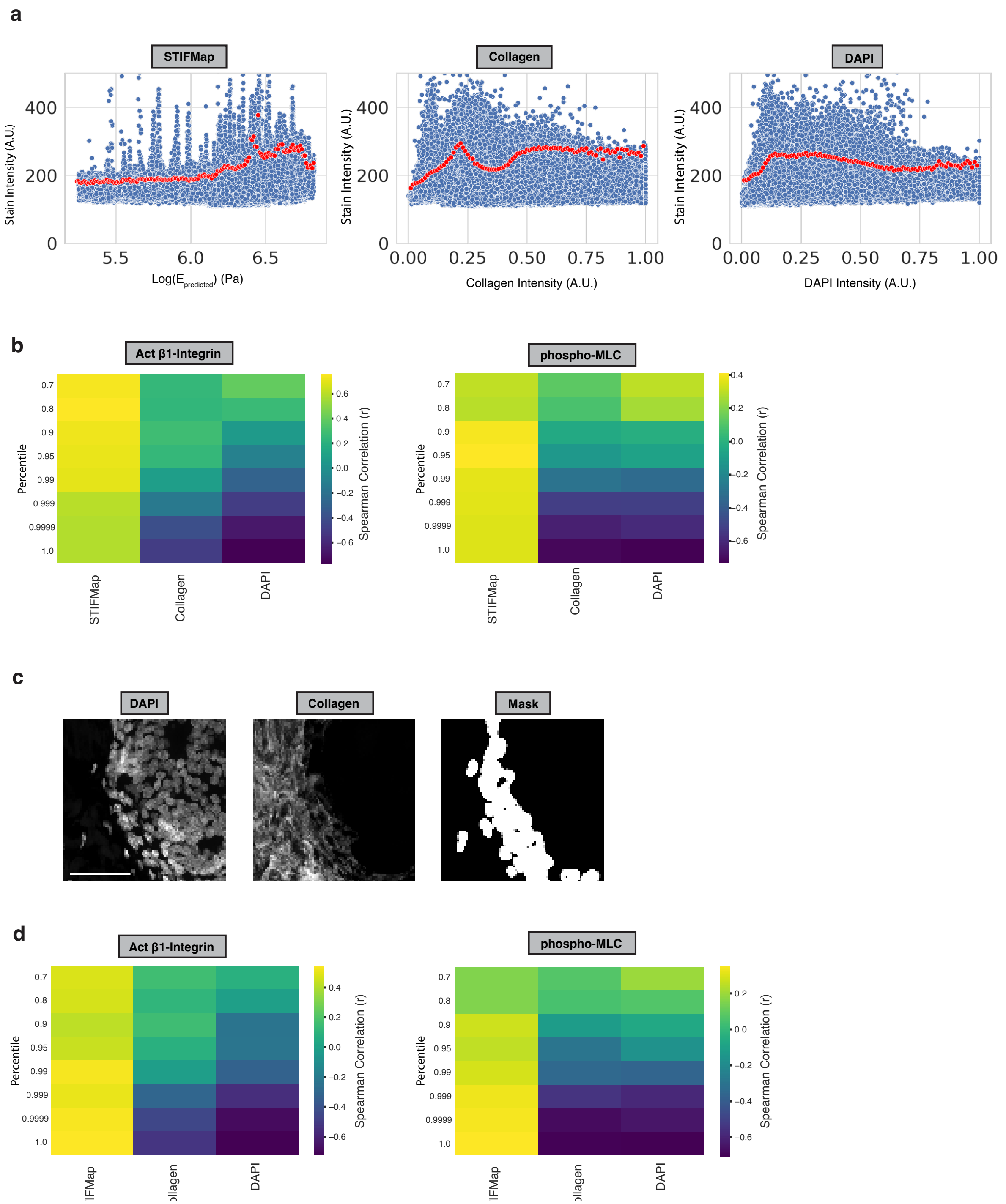

Supplementary Figure 4. Imaging Sensitivity Analysis. a, Scatterplots of stain intensity vs predicted stiffness (left), collagen intensity (middle), or DAPI intensity (right) shown for all pixels (blue) or aggregated to show the 99th percentile of stain intensity for each percentile of the indicated independent variable (red) for the representative pMLC stain shown in Fig. 3c. Aggregating data into percentiles is necessary to limit the influence of image regions where cells

are not interacting with the ECM. **b,** Sensitivity analysis of the average Spearman correlation coefficient as shown in (**a**) for stain intensity compared to DAPI, collagen, and STIFMap depending on the stain threshold used. n = 60 FOVs from 10 patient tumor samples. **c,** Representative FOV indicating pixels that are at the interface between cells and collagen. **d,** Sensitivity analysis of the average Spearman correlation coefficient depending on the stain threshold used when only masked pixels are included. n = 60 FOVs from 10 patient tumor samples. Scale bar, 50 μm. Source data are provided as a Source Data file.

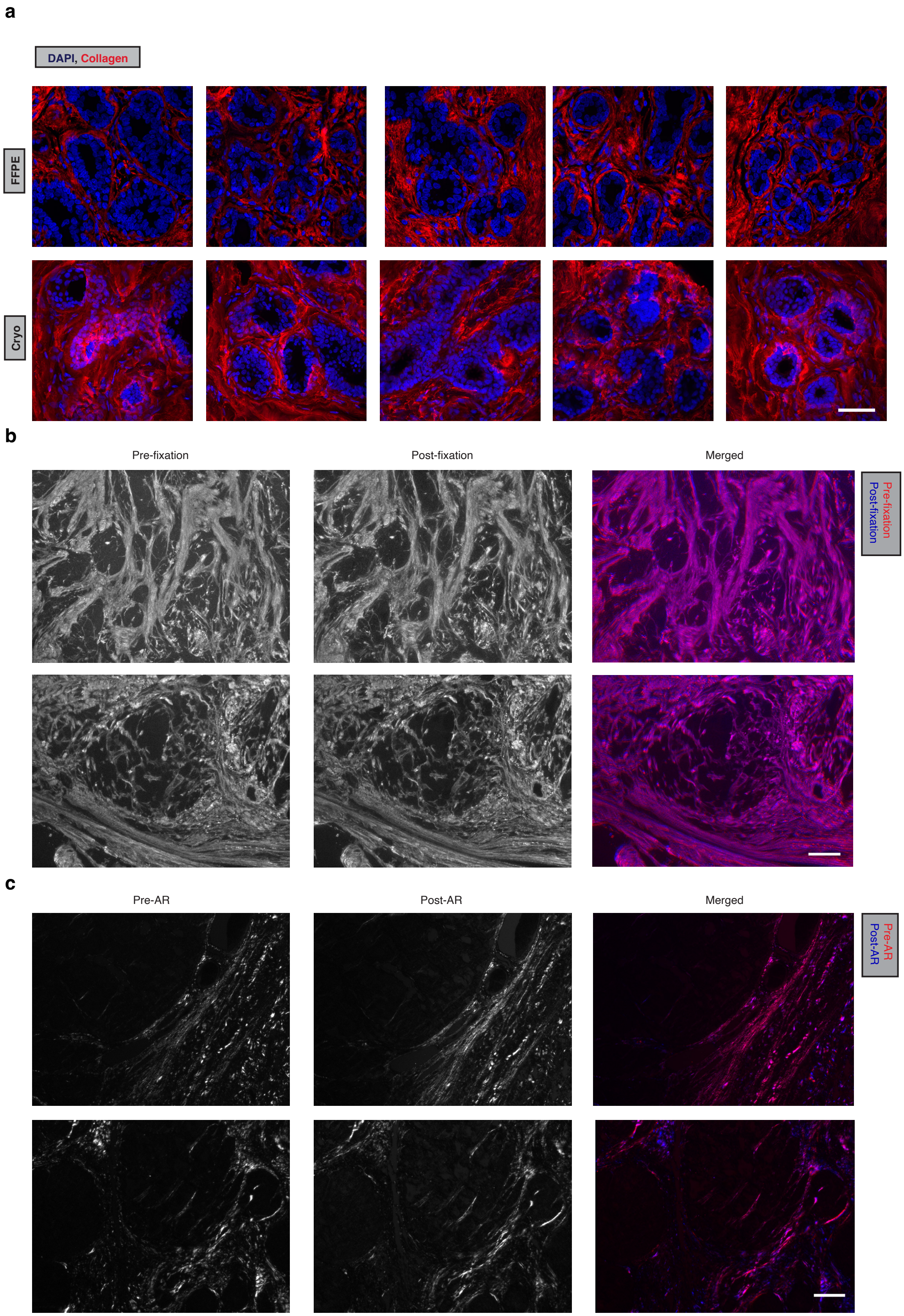
a
DAPI, Collagen
FFPE
Cryo
b
Pre-fixation
Post-fixation
Merged
Pre-fixation
Post-fixation
c
Pre-AR
Post-AR
Merged
Pre-AR
Post-AR

Supplementary Figure 5. Collagen Morphology Validation in FFPE Tissue. a, Five FOVs for an FFPE (top) or cryopreserved tissue (bottom) taken from the same patient stained with DAPI and CNA. Scale bar, 50μm. **b,** Representative images of collagen with CNA staining in cryopreserved tissues before and after 10% formalin-fixation for 1 hour. **c,** Representative images of collagen with PS red staining in FFPE tissues before and after antigen retrieval (AR). Red; pre and blue; post (b,c). Scale bar, 200 μm. Similar results for a, b and c were observed for 3 independent samples.

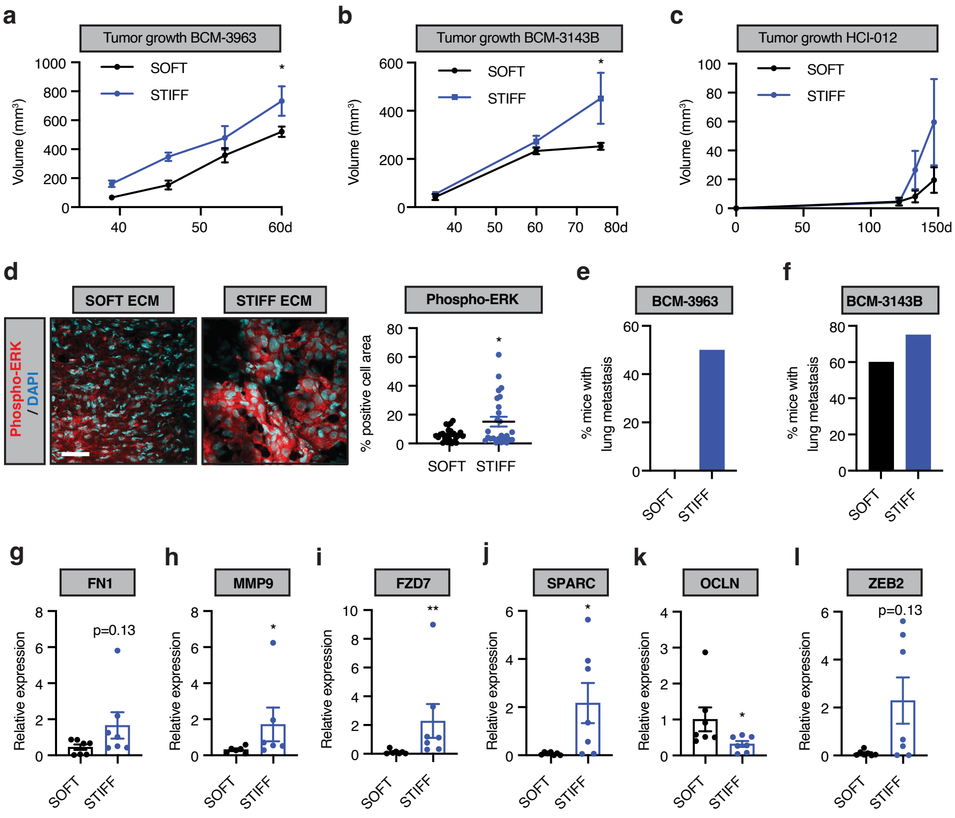

Supplementary Figure 6. A stiff stroma enhances mechanosignaling, tumor growth, metastasis, and mesenchymal gene expression in HER2-positive breast cancer PDXs. a-c, Graphs showing average tumor growth in SOFT and STIFF matrices for the HER2-positive PDX models indicated as determined by caliper measurement. SOFT and STIFF, n = 10 each for BCM-3963 and BCM-3143B, n = 4 each for HCI-012. p(SOFT vs STIFF; BCM-3963) = 0.0401. p(SOFT vs STIFF; BCM-3143B) = 0.0148. **d,** Representative images of immunofluorescence staining of phospho-ERK in SOFT and STIFF HER2-positive PDX tumors (left). Scale bar, 50 µm. Quantification of average phospho-ERK positive cell area for all HER2-positive PDX tumors (right). SOFT; n = 6, STIFF; n = 6. **e,f**, Percentage of mice bearing HER2-positive PDX tumors with SOFT and STIFF matrices presenting detectable lung metastases. SOFT and STIFF, n = 10 each for BCM-3963 and BCM-3143B. **g-l**, Graphs showing RT-PCR analysis of RNA extracted from HER2-positive PDX tumors with SOFT and STIFF matrices showing relative gene expression for the indicated mesenchymal and epithelial genes. SOFT; n = 7, STIFF; n = 7. p(SOFT vs STIFF; MMP9) = 0.0411. p(SOFT vs STIFF; FZD7) = 0.0023. p(SOFT vs STIFF; SPARC) = 0.0379. All graphs are presented as mean +/- S.E.M. Statistical tests used were two-sided Mann-

Whitney U test (**g-l**), two-sided unpaired *t*-test (**d**) and two-way ANOVA (with Bonferroni's multiple comparisons test) (**a-c**). *P<0.03, **P<0.002, ***P<0.0002, ns=non-significant. Source data are provided as a Source Data file.

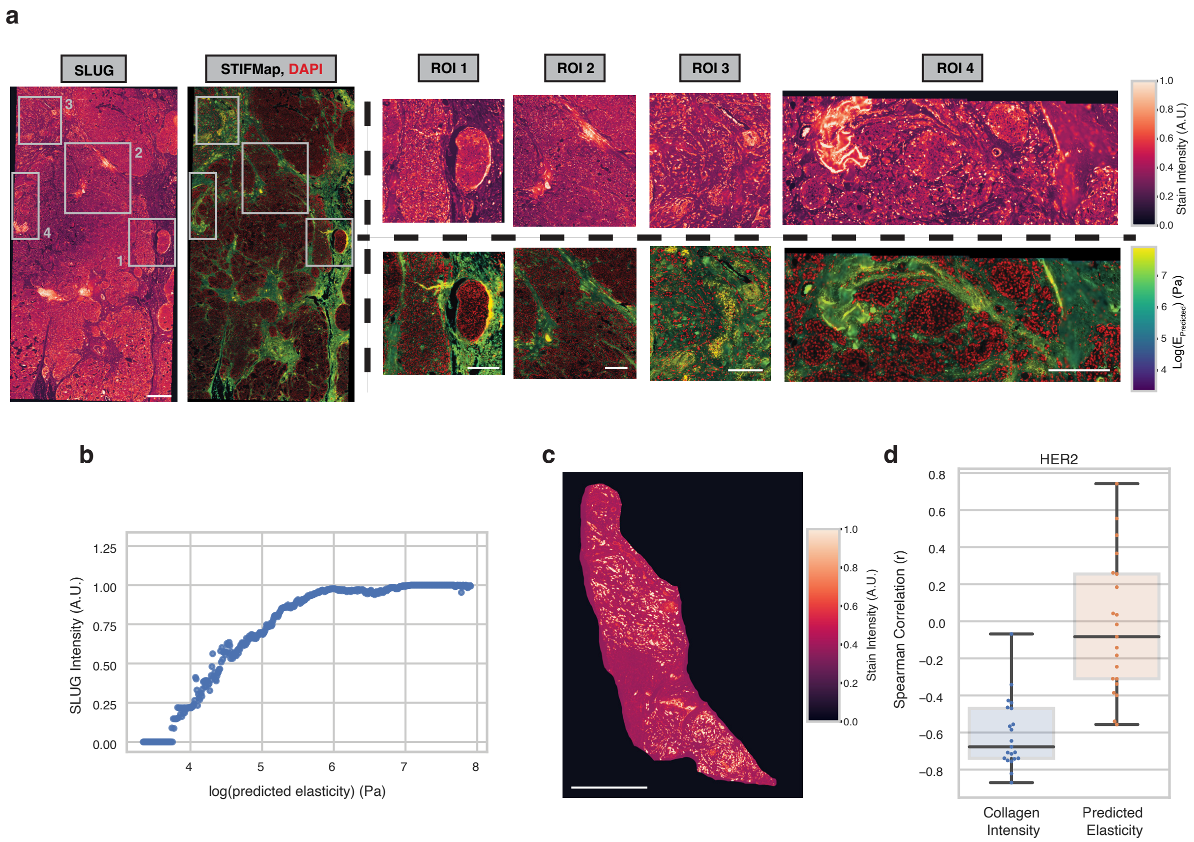

Supplementary Figure 7. Additional Patient EMT Staining Data. a, Representative WSI and ROIs for immunofluorescence staining of SLUG in a TNBC sample. 6 patient tumor samples were imaged. Scale bar (WSI), 100 µm. Scale bar (ROIs), 50 µm. **b,** Quantification of the 99th percentile of SLUG staining intensity for each percentile of predicted matrix elasticity for the image shown in (**a**). **c,** Representative HER2 stain from HER2+ breast cancer cohort (21 patient samples total). Scale bar, 1 mm. **d,** Correlation between HER2 intensity and either collagen intensity or predicted stiffness in the HER2+ cohort. n = 21 patient tumor samples. Boxes denote 25th to 75th percentile with median line. Whiskers mark the minima and the maxima excluding outliers. Source data are provided as a Source Data file.
