# Supplementary material for: A convolutional neural network STIFMap reveals associations between stromal stiffness and EMT in breast cancer
Source: Nat Commun. 2023 Jun 15;14:3561. doi: 10.1038/s41467-023-39085-1 (PMC10272194; doi:10.1038/s41467-023-39085-1)
Supplement: Supplementary file 3 — Reporting Summary [file 41467_2023_39085_MOESM3_ESM.pdf]

nature portfolio

Corresponding author(s): Valerie Weaver

Last updated by author(s): May 17, 2023

Reporting Summary

Nature Portfolio wishes to improve the reproducibility of the work that we publish. This form provides structure for consistency and transparency in reporting. For further information on Nature Portfolio policies, see our Editorial Policies and the Editorial Policy Checklist.

Statistics

For all statistical analyses, confirm that the following items are present in the figure legend, table legend, main text, or Methods section.

n/a	Confirmed	
☐	☒	The exact sample size (*n*) for each experimental group/condition, given as a discrete number and unit of measurement
☐	☒	A statement on whether measurements were taken from distinct samples or whether the same sample was measured repeatedly
☐	☒	The statistical test(s) used AND whether they are one- or two-sided<br>*Only common tests should be described solely by name; describe more complex techniques in the Methods section.*
☐	☒	A description of all covariates tested
☐	☒	A description of any assumptions or corrections, such as tests of normality and adjustment for multiple comparisons
☐	☒	A full description of the statistical parameters including central tendency (e.g. means) or other basic estimates (e.g. regression coefficient) AND variation (e.g. standard deviation) or associated estimates of uncertainty (e.g. confidence intervals)
☐	☒	For null hypothesis testing, the test statistic (e.g. *F*, *t*, *r*) with confidence intervals, effect sizes, degrees of freedom and *P* value noted<br>*Give P values as exact values whenever suitable.*
☒	☐	For Bayesian analysis, information on the choice of priors and Markov chain Monte Carlo settings
☒	☐	For hierarchical and complex designs, identification of the appropriate level for tests and full reporting of outcomes
☐	☒	Estimates of effect sizes (e.g. Cohen's *d*, Pearson's *r*), indicating how they were calculated

Our web collection on statistics for biologists contains articles on many of the points above.

Software and code

Policy information about availability of computer code

Data collection	AFM data was collected using Asylum's MFP-3D AFM software (v16) based in Igor. Confocal images were collected on either a Leica TCS SP5 Confocal miscrocope, a Nikon SoRa Spinning Disk microscope or an inverted Eclipse Ti-E Nikon Microscope with CSU-X1 spinning disk confocal. RNAseq data was collected on an Illumina NovaSeq6000.
Data analysis	All code necessary to train the STIFMap models, predict tissue stiffness, and pseudocolor histology images according to STIFMap predictions is available via https://github.com/cstashko/STIFMaps and and Zenodo under the DOI: 10.5281/Zenodo.78892270 https://doi.org/10.5281/zenodo.7882270. Full code and a complete pipeline for AutoAFM acquisition is available via https://github.com/cstashko/AutoAFM.

For manuscripts utilizing custom algorithms or software that are central to the research but not yet described in published literature, software must be made available to editors and reviewers. We strongly encourage code deposition in a community repository (e.g. GitHub). See the Nature Portfolio guidelines for submitting code & software for further information.

Data

Policy information about availability of data

All manuscripts must include a data availability statement. This statement should provide the following information, where applicable:

- Accession codes, unique identifiers, or web links for publicly available datasets
- A description of any restrictions on data availability
- For clinical datasets or third party data, please ensure that the statement adheres to our policy

The histology images utilized to train the STIFMaps model, the trained models themselves, and the training logs and statistics are available via https://

data.mendeley.com/datasets/vw2bb5jy99/2. STIFMap predictions is available via https://github.com/cstashko/STIFMaps and Zenodo under the DOI: 10.5281/Zenodo.78892270 https://doi.org/10.5281/zenodo.7882270. Raw RNAseq data is available through GEO series access number GSE179983. We provide a source data file with all the relevant raw data that was used to generate every figure.

Human research participants

Policy information about studies involving human research participants and Sex and Gender in Research.

Reporting on sex and gender	All patients participating in this study were female by birth.
Population characteristics	All patients were treatment naïve when clinical samples were collected. Only samples of invasive ductal carcinoma that were centrally verified as HER2+ or TNBC were analyzed. For the clinical cohort of HER2+ samples, untreated biopsies were compared, and metastatic recurrence data was collected.
Recruitment	All human breast tissue specimens were collected prospectively from consenting female patients (all patients provided written informed consent prior to surgery) undergoing surgery at the University of California, San Francisco, (UCSF) or Duke University Medical Center between 2010 and 2020. Patients were not compensated for consenting to tissue donation.
Ethics oversight	Institutional Review Board Protocol #10-03832, approved by the UCSF Committee of Human Resources and the Duke University IRB (Pro00054515).

Note that full information on the approval of the study protocol must also be provided in the manuscript.

Field-specific reporting

Please select the one below that is the best fit for your research. If you are not sure, read the appropriate sections before making your selection.

[x] Life sciences [ ] Behavioural & social sciences [ ] Ecological, evolutionary & environmental sciences

For a reference copy of the document with all sections, see nature.com/documents/nr-reporting-summary-flat.pdf

Life sciences study design

All studies must disclose on these points even when the disclosure is negative.

Sample size	For neural network training, sample size was determined to be on the same scale as previously reported deep learning applied to regression tasks. Mouse cohort size was determined based on power analysis of cohort sizes previously shown to demonstrate mechano-responsive differences in tumor growth and metastasis.
Data exclusions	AFM data points were excluded if no clear contact point could be established by the researchers, thereby preventing their inclusion as 'ground truth' tissue stiffness measurements.
Replication	Tissue elasticity predictions made using STIFMaps were validated via staining for the mechano-regulated markers phospho-MLC and activated beta1 integrin, as described in the manuscript. By working with three different PDX models, we ensured that mechanoresponsiveness was robust beyond a patient-specific effect. Sample sizes are indicated in corresponding figure sizes and methods.
Randomization	Mice were randomly assigned between soft and stiff PDX groups. A random subset of responsive and nonresponsive patient samples was selected from the HER2 cohort.
Blinding	After PDX injection, researchers were blinded as to which mice received soft or stiff tumors until RNAseq results were obtained. Researchers were blinded as to whether HER2 tumors were chemoresponsive or nonresponsive until heterogeneity statistics were generated.

Reporting for specific materials, systems and methods

We require information from authors about some types of materials, experimental systems and methods used in many studies. Here, indicate whether each material, system or method listed is relevant to your study. If you are not sure if a list item applies to your research, read the appropriate section before selecting a response.

Materials & experimental systems

n/a	Involved in the study	
☐	☒	Antibodies
☒	☐	Eukaryotic cell lines
☒	☐	Palaeontology and archaeology
☐	☒	Animals and other organisms
☒	☐	Clinical data
☒	☐	Dual use research of concern

Methods

n/a	Involved in the study	
☒	☐	ChIP-seq
☒	☐	Flow cytometry
☒	☐	MRI-based neuroimaging

Antibodies

Antibodies used	Immunofluorescence was performed using the following specific antibodies: phospho-FAK (Y397) (Cell Signaling Technology, Cat. #: 8556, 1:200), phospho-p44/42 MAPK (ERK1/2) (T202/Y204) (Cell Signaling Technology, Cat. #: 9101, 1:200), Integrin β1, activated (Sigma-Aldrich, clone HUTS-4, Cat. #: MAB2079Z, 1:400), phospho-Myosin Light Chain 2 (Ser19) (Cell Signaling Technology, Cat. #: 3671, 1:200), yes-activated protein (YAP) (Santa Cruz, Cat. #: sc-15407, 1:200), SLUG (Cell Signaling Technology, clone C19G7, Cat. #: 9585, 1:200), ZEB1 (Cell Signaling Technology, clone E2G6Y, Cat. #: 70512), Twist1 (Abcam, Cat. #: ab50887, 1:500) and ErbB2 / HER2 antibody (Abcam, clone 3B5, Cat. #: ab16901)
Validation	Antibodies were validated by titrating their concentration within an order of magnitude of the manufacturer's recommendation and using appropriate positive and negative controls to ensure specificity.

Animals and other research organisms

Policy information about studies involving animals; ARRIVE guidelines recommended for reporting animal research, and Sex and Gender in Research

Laboratory animals	NOD/SCID mice were purchased from Jackson Laboratories and sacrificed in accordance with IACUC and LARC protocols twelve weeks after PDX injection or at maximal tumor burden.
Wild animals	The study did not involve wild animals
Reporting on sex	All animal data was collected from female mice.
Field-collected samples	The study did not include samples collected from the field.
Ethics oversight	Animal husbandry and all procedures on mice were carried out in Laboratory Animal Resource Center (LARC) facilities at UCSF Parnassus in accordance with the guidelines stipulated by the Institutional Animal Care Use Committee (IACUC) protocols, #AN133001 and #AN179766, which adhere to the NIH Guide for the Care and Use of Laboratory Animals.

Note that full information on the approval of the study protocol must also be provided in the manuscript.
